# Analysis of the Risk Factors Associated With Obstructive Sleep Apnea Syndrome in Chinese Children

**DOI:** 10.3389/fped.2022.900216

**Published:** 2022-06-27

**Authors:** Ling Xiao, Shuping Su, Jia Liang, Ying Jiang, Yan Shu, Ling Ding

**Affiliations:** ^1^Department of Otolaryngology, Children's Hospital of Chongqing Medical University, Chongqing, China; ^2^National Clinical Research Center for Child Health and Disorders, Chongqing, China; ^3^Ministry of Education Key Laboratory of Child Development and Disorders, Chongqing, China; ^4^China International Science and Technology Cooperation Base of Child Development and Critical Disorders, Chongqing, China; ^5^Chongqing Key Laboratory of Pediatrics, Chongqing, China

**Keywords:** obstructive sleep apnea, risk factor, child, adenoid hypertrophy, tonsil hypertrophy

## Abstract

**Objective:**

The present study was developed to explore risk factors related to the incidence and severity of obstructive sleep apnea syndrome (OSAS) in children.

**Methods:**

The present study enrolled pediatric patients who admitted to our department for snoring and/or open-mouth breathing. All children completed a questionnaire and underwent physical examination and polysomnography (PSG). The cases were separated into OSAS and primary snoring (PS) groups. Factors associated with these two groups were analyzed, with risk factors significantly associated with OSAS then being identified through logistic regression analyses. OSAS was further subdivided into mild, moderate, and severe subgroups, with correlations between risk factors and OSAS severity then being analyzed.

**Results:**

In total, 1,550 children were included in the present study, of which 852 and 698 were enrolled in the OSAS and PS groups. In univariate analyses, obesity, family passive smoking, a family history of snoring, allergic rhinitis, asthma, adenoid hypertrophy, and tonsil hypertrophy were all related to pediatric OSAS (*P* < 0.05). In a multivariate logistic regression analysis, adenoid hypertrophy (OR:1.835, 95% CI: 1.482–2.271) and tonsil hypertrophy (OR:1.283, 95% CI:1.014–1.622) were independently associated with the risk of pediatric OSAS (*P* < 0.05). Stratification analyses revealed that OSAS incidence increased in a stepwise manner with increases in adenoid and tonsil grading (*P* < 0.01). Correlation analyses revealed that adenoid hypertrophy and tonsilar hypertrophy were not significantly associated with OSAS severity (*r* = 0.253, 0.069, respectively, *P* < 0.05), and tonsil and adenoid size were no correlation with obstructive apnea-hypopnea index (OAHI) (*r* = 0.237,0.193, respectively, *P* < 0.001).

**Conclusion:**

Obesity, family passive smoking, a family history of snoring, allergic rhinitis, asthma, tonsil hypertrophy, and adenoid hypertrophy may be potential risk factors for pediatric OSAS. Adenoid hypertrophy and tonsil hypertrophy were independently related to the risk of pediatric OSAS, with OSAS incidence increasing with the size of the adenoid and tonsil, while the severity of OSAS is not parallel related to the adenoid or tonsil size.

## Introduction

Pediatric obstructive sleep apnea syndrome (OSAS) corresponds to a series of pathophysiological alterations as a result of the frequent total or partial of obstruction of the airway during sleep that can interfere with normal sleep structure and ventilation in children (1). The clinical manifestations of primary snoring (PS) and OSAS both include snoring, which is a form of sleep-disordered breathing (SDB) in children, but OSAS is additionally characterized by a SaO_2_ decline and/or hypercapnia, while PS is not associated with OSA, hypoxemia, altered sleep structure, or any abnormalities of alveolar ventilation or oxygen levels. The 2012 American Academy of Pediatrics (AAP) guidelines estimate that about 11% of children in the United States between the ages of 1 and 9 years snore, and that 2–3% of all children or 50% of snorers have OSAS (2), with data from 2010 similarly suggesting a pediatric OSAS prevalence rate of 4.8% in Hong Kong, China (3). while the reported childhood incidence rate of PS is 6.1–15.5% (4, 5). When not diagnosed and treated in a timely manner, pediatric OSAS can contribute to severe complications including growth and developmental retardation, learning disorders, neurocognitive impairment, maxillofacial dysplasia, hypertension, pulmonary hypertension, metabolic disorders, endocrine disorders, and higher events of cardiovascular events that persist into adulthood (6). OSAS is the most serious disease in children with sleep disordered breathing. Given its high prevalence and potential for serious long-term complications, OSAS has attracted increasing attention from parents and society as a whole.

It is vital that risk factors associated with OSAS incidence and severity be defined in order to better screen for and manage this disease. Detecting these risk factors at an early time point can facilitate appropriate timely intervention, thereby preventing the progression of pediatric OSAS. Wang et al. (7) found that gender, obesity, the adenoid A/N ratio, and tonsil size were all associated with the severity of obstructive sleep apnea, while Zhang et al. (8) found obesity to be an important factor affecting the severity of OSAS. Kang et al. (9) determined that male gender and obesity increased the risk of pediatric obstructive sleep apnea, and discovered that the apnea-hypopnea index (AHI) in male adolescents (15–18 years old) was higher than that in young boys (3–6 years old). However, the only large-scale studies exploring pediatric OSAS risk factors in China to date were conducted in Beijing Children's Hospital and Fujian Children's Hospital (10, 11). In contrast, data in the present study are derived from the Affiliated Children's Hospital of Chongqing Medical University in Chongqing, which is one of the four municipalities directly under the central government. It is the third children's Hospital in China, and the largest diagnosis and treatment center for children's otolaryngology and head and neck diseases in Southwest China. This study was developed to explore risk factors related to the incidence and severity of pediatric OSAS through questionnaires and surveys in an effort to provide guidelines for patient diagnosis and treatment.

## Materials and Methods

### Subjects and Study Design

The present study prospectively enrolled pediatric patients who admitted to our department for snoring and/or open-mouth breathing between June 2020 and January 2021. Patients were eligible for inclusion if they were <18 years of age, had undergone polysomnography (PSG) and electronic nasopharyngoscopy, and their parents or guardians had completed the questionnaire. Patients were excluded if they had a history of adenotonsillectomy, craniofacial abnormalities, neuromuscular disease, or comorbidities including Crouzon syndrome, Down syndrome, or Pierre Robin sequence. Children previously treated for OSAS using medications (e.g., intranasal steroids or leukotriene receptor antagonists) were also excluded. Informed consent of parents or guardians was required for the completion of the questionnaire survey. The institutional review board of the Children's Hospital of Chongqing Medical University approved this study.

Through a combination of extensive literature review and clinical experience, our research group independently designed a questionnaire to collect data pertaining to possible OSAS-related risk factors. Parameters included in this questionnaire included age, gender, obesity status, parental educational level, mode of delivery, term or pre-term birth, feeding mode, family history of passive smoking, family history of snoring, allergic rhinitis, asthma, tonsillar hypertrophy, and adenoid hypertrophy. Obesity was defined based on the presence of a body mass index (BMI) >95% of the average for children of the same age and sex. Pre-term birth was defined by birth at a gestational age <37 weeks. Breastfeeding was defined by exclusive breastfeeding for at least 4 months following birth. A family history of passive smoking was considered to be present when any family member(s) living in the same residence as the child reported smoking 1+ cigarettes per day. A family history of snoring was considered positive when parents reported that the parents and/or siblings of the child had a history of snoring. Asthma and allergic rhinitis (AR) were considered positive when parents reported that a child had been diagnosed with these conditions by a physician. The contents of the questionnaire were answered by the parents or guardians.

### Measurements

Prior to PSG analysis, all patients underwent physical examinations that included measurements of weight and height. For tonsil inspection, children were directed to open their mouth, and a tongue depressor was gently placed on the first two-thirds of the tongue in front of the eversion nipple, so as to prevent the obstruction and medial displacement of the tonsils. Tonsil sizes were graded from I to IV (12), with grade I corresponding to confinement to the tonsillar fossa, Grade II corresponding to protrusion from the palatoglossal arch and occupying 50% of the pharyngeal space, Grade III corresponding to protrusion from the tonsillar fossa and occupying 75% of the pharyngeal space, and Grade IV corresponding to the blocking of the pharyngeal space by the bilateral tonsils, which were nearly folded. Tonsillar hypertrophy was defined by Grade III-IV tonsils with corresponding clinical symptoms (12).

All children underwent electronic nasopharyngoscopy, with the local nasal mucosa being shrunken *via* the application of ephedrine nasal drops after which the electronic nasopharyngoscope was inserted into the nasal cavity *via* the middle anterior nostril and extended to the posterior portion of the nasal cavity *via* the wider middle and/or lower nasal passages to analyze adenoid sizing, which was graded as follows (13): adenoid enlargement-associated obstruction of the posterior nostrils by ≤25%, 26–50%, 51–75%, and 76–100% was defined as Grade I, II, III, and IV, respectively. Adenoid hypertrophy was defined by Grade III or IV adenoids with corresponding clinical symptoms (13).

Polysomnography examinations were conducted in the sleep center using a PSG System (Alice 6 LDx, USA). Parents remained present in the room with children during testing, with each child being monitored for a minimum of 8 h. Children were subjected to electroencephalography (EEG), electrooculography (EOG), electromyography (EMG), electrocardiography, airflow measurements, respiratory effort analysis, and monitor blood oxygen saturation. Sleep stages were defined as per the American Academy of Sleep Medicine manual (14). Those children with an obstructive apnea-hypopnea index (OAHI) value of ≥1 were defined as having OSAS (1)^.^Children were then separated into groups based upon the observed sleep disturbance severity levels as follows: an OSAS group (OAHI ≥1) and a PS group (OAHI < 1 with a clinical history of snoring). Children in the OSAS group were then further subdivided into those with mild (1< OAHI ≤5), moderate (5< OAHI ≤10), and severe disease (OAHI>10) (1).

### Statistical Analysis

The normality of continuous data distributions was tested using the Kolmogorov-Smirnov test. If the measurement data were normally distributed, *t*-tests were used to compare the two groups and data were reported as mean ± standard deviation. If data were not normally distributed, they were compared *via* Mann-Whitney *U*-tests and reported as median (first quartile—third quartile). The classification/graded data were reported as frequencies with percentages, and univariate analyses of categorical data were conducted using chi-square tests. Then, those factors that were found to be significant in univariate analyses were incorporated into a multivariate logistic regression analysis. Spearman correlation analyses were used to compare associations between variables and the severity of OSAS. And analysis the relative disease severity in obese and non-obese children in the OSAS group. All analyses were performed using the SPSS 21.0 statistical software, with *P* < 0.05 as the threshold of significance.

## Results

### General Characteristics Comparison Between the OSAS and PS Groups

In total, questionnaires were completed for 1,550 children, including 852 in the OSAS group [517 male, 335 female; age: 5.0 (3.9–6.4 years)] and 698 patients in the PS group [405 male, 293 females; age: 5.1 (4.1–6.6 years)]. There were no significant differences between groups in age or gender distributions (*P* > 0.05). The incidence of obesity, household passive smoking, household snoring history, allergic rhinitis, asthma, adenoid hypertrophy, and tonsil hypertrophy was increased in the OSAS group relative to the PS group (*P* < 0.05), indicating that these were potential risk factors for OSAS in children. However, there were no significant differences in parental education level, mode of delivery, feeding mode, and pre-maturity between the two groups (Table 1).

**Table 1:** Patient characteristics in the OSAS and PS groups.

**Parameters**		***n***	**OSAS group**	**PS group**	**X^**2**^/Z**	***P*-Value**
Total		1,550	852 (55.0%)	698 (45.0%)		
Gender	Male	922 (59.5%)	517 (60.7%)	405 (58.0%)	1.125	0.289
	Female	628 (40.5%)	335 (39.3%)	293 (42.0%)		
Age (years), median (first quartile—third quartile)		1,550	5.0 (3.9–6.4)	5.1 (4.1–6.6)	1.736	0.083
Age section	Toddler (age 1–3)	112 (7.2%)	72 (8.4%)	40 (5.7%)	4.240	0.237
	Pre-school (age 3–6)	963 (62.1%)	522 (61.3%)	441 (63.2%)		
	School (age 6–12)	455 (29.4%)	247 (29.0%)	208 (29.8%)		
	Adolescence (age 12–18)	20 (1.3%)	11 (1.3%)	9 (1.3%)		
Obesity	Yes	188 (12.1%)	117 (13.7%)	72 (10.3%)	4.185	0.041*
	No	1,362 (87.9%)	735 (83.3%)	626 (89.7%)		
Father's education	Junior high school or below	198 (12.8%)	116 (13.6%)	82 (11.7%)	1.605	0.448
	High school	378 (24.4%)	211 (24.8%)	167 (24.0%)		
	University or above	974 (62.8%)	525 (61.6%)	449 (64.3%)		
Mother's education	Junior high school or below	210 (13.6%)	118 (13.8%)	92 (13.2%)	0.269	0.847
	High school	320 (20.6%)	178 (20.9%)	142 (20.3%)		
	University or above	1,020 (65.8%)	556 (65.3%)	464 (66.5%)		
Mode of delivery	Natural birth	888 (57.3%)	475 (55.8%)	413 (59.2%)	1.832	0.176
	Cesarean delivery	662 (42.7%)	377 (44.2%)	285 (40.8%)		
Pre-term birth	Yes	97 (6.3%)	54 (6.3%)	43 (6.2%)	0.021	0.886
	No	1,453 (93.7%)	798 (93.7%)	655 (93.8%)		
Feeding mode	Breast feeding	1,022 (66.0%)	573 (63.0%)	449 (64.3%)	1.473	0.497
	Mixed feeding	315 (20.3%)	167 (19.6%)	148 (21.2%)		
	Artificial feeding	213 (13.7%)	112 (14.4%)	101 (14.5%)		
Family passive smoking	Yes	836 (53.9%)	481 (56.5%)	355 (50.9%)	4.836	0.028*
	No	714 (46.1%)	371 (43.5%)	343 (49.1%)		
Family history of snoring	Yes	1,026 (66.2%)	577 (67.7%)	439 (62.9%)	3.962	0.047*
	No	524 (33.8%)	275 (32.3%)	259 (37.1%)		
Allergic rhinitis	Yes	314 (20.3%)	189 (27.2%)	125 (17.9%)	4.430	0.037
	No	1,236 (79.7%)	663 (77.8%)	573 (82.1%)		
Asthma	Yes	141 (9.1%)	89 (10.4%)	52 (7.4%)	4.165	0.041*
	No	1,409 (90.9%)	763 (89.6%)	646 (92.6%)		
Adenoid hypertrophy	Yes	1,005 (64.8%)	605 (71.0%)	400 (57.3%)	31.60	0.000*
	No	545 (35.2%)	247 (29.0%)	298 (42.7%)		
Tonsil hypertrophy	Yes	1,153 (74.4%)	652 (76.5%)	501 (71.8%)	4.542	0.033*
	No	397 (25.6%)	200 (23.5%)	197 (28.2%)		

^*^P <0.05.

### Univariate Analysis and Multivariate Logistic Regression Analysis of OSAS-Related Risk Factors

The univariable logistic regression analysis showed that the obesity, family passive smoking, family history of snoring, allergic rhinitis, asthma, adenoid hypertrophy, and tonsil hypertrophy were the potential risk factors for pediatric OSAS (*P* < 0.05). Those parameters that were significantly different between groups in univariate analyses were further incorporated into a multivariate logistic regression analysis which revealed that adenoid hypertrophy (OR = 1.835, 95% CI: 1.482–2.271) and tonsil hypertrophy (OR = 1.283, 95% CI: 1.014–1.622) were independent risk factors associated with the incidence of pediatric OSAS (*P* < 0.05). Nevertheless, obesity, family passive smoking, family history of snoring, allergic rhinitis, and asthma were not independent risk factors for pediatric OSAS (Table 2).

**Table 2:** Univariable and multivariate logistic regression analysis of pediatric OSAS-related risk factors.

**Parameters**	**Univariate analysis**				**Multivariate analysis**						
	***P*-Value**	**Exp (B)**	**95% CI**		**B**	**S.E**.	**Wald**	***P*-Value**	**Exp (B)**	**95% CI**	
Obesity	0.041*	1.384	1.013	1.891	0.248	0.164	2.289	0.130	1.281	0.929	1.766
Family passive smoking	0.028*	1.253	1.025	1.531	0.187	0.105	3.200	0.074	1.206	0.982	1.480
Family history of snoring	0.047*	1.238	1.003	1.528	0.171	0.109	2.442	0.118	1.187	0.957	1.470
Allergic rhinitis	0.038*	1.307	1.016	1.681	0.247	0.135	3.341	0.068	1.281	0.982	1.670
Asthma	0.042*	1.449	1.013	2.072	0.327	0.192	2.913	0.088	1.387	0.953	2.020
Adenoid hypertrophy	0.000*	1.825	1.478	2.253	0.607	0.109	31.043	0.000*	1.835	1.482	2.271
Tonsillar hypertrophy	0.033*	1.282	1.020	1.611	0.249	0.120	4.321	0.038*	1.283	1.014	1.622

SE, standard error; OR, odds ratio; CI, confidence interval; *P <0.05.

### The Relationship Between Adenoid and Tonsil Hypertrophy and the Incidence of OSAS

Next, a more detailed analysis of the relationship between OSAS incidence and adenoid and tonsilar hypertrophy was conducted. Relative to grade I adenoid hypertrophy, the OR values for grade II, III, and IV adenoid hypertrophy were 2.285 (95% CI: 1.713–3.049), 3.006 (95% CI: 2.140–4.221), and 3.554 (95% CI: 2.514–5.024), respectively. Relative to grade I tonsillar hypertrophy, the OR values for grade II, III, and IV tonsillar hypertrophy were 1.478 (95% CI: 1.167–1.870), 1.551 (95% CI: 1.115–2.157), 1.631 (95% CI: 1.174–2.267), respectively. Chi-squared tests revealed that OSAS incidence rose in a stepwise manner with increasing adenoid and tonsillar hypertrophy grades (*P* < 0.01), suggesting that the larger these organs, the greater the risk of pediatric OSAS development (Table 3).

**Table 3:** The tonsil and adenoid sizes of children in the OSAS and PS groups.

**Parameters**		**OSAS group** **(*n* = 852)**	**PS group (*n* = 698)**	***P*-Value**	**OR value**	**95% CI**	
Adenoid	Grade I	114 (13.4%)	150 (21.5%)		1.00		
hypertrophy	Grade II	133 (15.6%)	148 (21.2%)	0.000*	2.285	1.713	3.049
	Grade III	370 (43.4%)	313 (44.8%)	0.000*	3.006	2.140	4.221
	Grade IV	235 (27.6%)	87 (12.5%)	0.009*	3.554	2.514	5.024
Tonsillar	Grade I	99 (11.6%)	100 (14.3%)		1.00		
hypertrophy	Grade II	101 (11.9%)	97 (13.9%)	0.001*	1.478	1.167	1.870
	Grade III	329 (38.6%)	301 (43.1%)	0.009*	1.551	1.115	2.157
	Grade IV	323 (37.9%)	200 (28.7%)	0.004*	1.631	1.174	2.267

^*^P <0.05.

### Correlations Between Risk Factors and the Severity of Pediatric OSAS

We further stratified OSAS patients into mild, moderate, and severe disease subgroups (*n* = 603, 157, and 92, respectively). Correlation analyses revealed adenoid hypertrophy was weak associated with OSAS severity (*r* = 0.253, *P* < 0.05), and tonsilar hypertrophy, obesity, allergic rhinitis were no associated with OSAS severity (*r* = 0.069, 0.070, and 0.127, respectively, *P* < 0.05), while family history of passive smoking, family history of snoring, asthma, and OSAS severity were not correlated with such severity (*P* > 0.05) (Table 4). Further correlation analyses revealed that tonsil size have weak relationship with the OSAS severity (*r* = 0.286, *P* < 0.001), while there was no correlation between adenoid size and OSAS severity (*r* = 0.194, *P* < 0.001) (Table 5). And tonsisl size and adenoid size were no correlation with OAHI (*r* = 0.237, 0.193, respectively, *P* < 0.001).

**Table 4:** Spearman correlation analyses of the relationships between risk factors and the severity of pediatric OSAS.

**Parameters**	**Mild OSAS**	**Moderate OSAS**	**Severe OSAS**	**Correlation co-efficient *r* value**	***P*-Value**
Obesity (*n* = 117)	74 (63.2%)	25 (21.4%)	18 (15.4%)	0.070	0.042*
Family passive smoking (*n* = 481)	358 (74.4%)	75 (15.6%)	48 (10.0%)	0.023	0.632
Family history of snoring (*n* = 577)	408 (70.7%)	111 (19.2%)	58 (10.1%)	0.005	0.883
Allergic rhinitis (*n* = 189)	154 (81.5%)	24 (12.7%)	11 (5.8%)	0.127	0.000*
Asthma (*n* = 89)	74 (83.1%)	11 (12.4%)	4 (4.5%)	0.031	0.551
Adenoid hypertrophy (*n* = 605)	384 (53.5%)	136 (22.5%)	85 (14.0%)	0.253	0.000*
Tonsillar hypertrophy (*n* = 652)	453 (69.5%)	115 (17.6%)	84 (12.9%)	0.069	0.044*

^*^P < 0.05.

**Table 5:** Spearman correlation analyses of the relationships between adenotonsillar hypertrophy and the severity of pediatric OSAS.

**Parameters**		**Mild OSAS** **(*n* = 603)**	**Moderate OSAS** **(*n* = 157)**	**Severe OSAS** **(*n* = 92)**	**Correlation co-efficient *r* value**	***P*-Value**
Adenoid hypertrophy	Grade I	99 (16.4%)	15 (9.6%)	0 (0.0%)	0.286	<0.001
	Grade II	120 (19.9%)	6 (3.8%)	7 (7.6%)		
	Grade III	256 (42.5%)	76 (48.4%)	38 (41.3%)		
	Grade IV	128 (21.2%)	60 (38.2%)	47 (51.1%)		
Tonsillar hypertrophy	Grade I	89 (14.8%)	10 (6.4%)	0 (0.0%)	0.194	<0.001
	Grade II	61 (10.1%)	32 (20.4%)	8 (8.7%)		
	Grade III	261 (43.3%)	45 (28.6%)	23 (25%)		
	Grade IV	192 (31.8%)	70 (44.6%)	61 (66.3%)		

### Analysis of Relative Disease Severity in Obese and Non-obese Children in the OSAS Group

The children in the OSAS group were next divided into obese (*n* = 117) and non-obese (*n* = 753) groups. The grading of adenoid and tonsil sizes did not differ significantly between these two groups (*P* > 0.05). However, PSG parameters were significantly increased for obese patients relative to non-obese patients (*P* < 0.001) (Table 6).

**Table 6:** Analysis of disease severity in obese and non-obese children in the OSAS group.

		**Obese group** **(*n* = 117)**	**Non-obese group** **(*n* = 735)**	***P*-Value**
Adenoid	Grade I	14 (12.0%)	100 (13.6%)	0.062
hypertrophy	Grade II	18 (15.4%)	115 (15.6%)	
	Grade III	41 (35.0%)	329 (44.8%)	
	Grade IV	44 (37.6%)	191 (26.0%)	
Tonsillar	Grade I	15 (12.8%)	84 (11.4%)	0.740
hypertrophy	Grade II	17 (14.5%)	84 (11.4%)	
	Grade III	43 (36.8%)	286 (38.9%)	
	Grade IV	42 (35.9%)	281 (38.3%)	
OSAS severity	Mild	32 (27.4%)	571 (77.7%)	0.000
	Moderate	41 (35.0%)	116 (15.8%)	
	Severe	44 (37.6%)	48 (6.5%)	

## Discussion

Pediatric OSAS is a complex condition associated with a diverse array of risk factors and potential complications, with the resultant clinical presentation being shaped by a confluence of genetic, environmental, and lifestyle factors. Efforts to identify OSAS-related risk factors at an early time point are essential to improving patient outcomes. In prior reports, tonsillar hypertrophy and adenoid hypertrophy (7, 10, 11), as well as obesity (8, 15), were identified as critical factors associated with the development of OSAS in children. Other risk factors including asthma (16), pre-term birth (17, 18), being male (11), parental smoking (19, 20), habitual snoring (21), and ethnicity (22) have also been reported to be associated with OSAS incidence, but these findings are somewhat controversial. The purpose of the present study was to identify independent risk factors related to the clinical risk of OSAS by recruiting children of all ages through our clinic. This approach revealed that obesity, a family history of passive smoking, a family history of snoring, allergic rhinitis, asthma, adenoid hypertrophy, and tonsillar hypertrophy were all present at significantly higher rates among pediatric OSAS patients relative to PS patients, with adenoid hypertrophy and tonsillar hypertrophy being independently associated with the risk of OSAS incidence.

In this study, we found both adenoid hypertrophy and tonsillar hypertrophy to be independently related to pediatric OSAS risk, in line with other reports (7, 10). Adenotonsillar hypertrophy (ATH) has been thought to be the most critical risk factor associated with OSAS development among children at present. Tagaya et al. (23) discovered that adenoid grade and AHI were significantly correlated in pre-school-aged children, and that the influence of adenoid hypertrophy decreased from pre-school to schoolchildren, whereas tonsil size was largely unrelated to AHI values. Another study conducted by Chuang et al. (24) noted that disease severity was independently correlated with tonsil size and adenoid grade, and that tonsillar hypertrophy was the most influential factor for younger children, whereas adenoidal hypertrophy became more important at an older age. Both the adenoids and tonsils are lymphoid tissues, and the highest rates of hyperplasia occur from 3 to 6 years of age such that different adenoid and tonsil sizes can have a different impact on AHI values, explaining why some instances of adenoid hypertrophy and/or tonsil hypertrophy only lead to the occurrence of PS. Adenoid hypertrophy results in the obstruction of the posterior nostrils and the nasopharynx, while tonsillar hypertrophy results in profound pharyngeal stenosis and can obstruct airflow, thus contributing to OSAS development. First-line treatment for pediatric OSAS patients thus consists of tonsillectomy and adenoidectomy in children exhibiting clinical symptoms of ATH.

Our study revealed that the incidence of OSAS was positively associated with the degree of hypertrophy in the adenoids and tonsils, consistent with the findings of Shen et al. (10). Specifically, the incidence of OSAS in individuals with grade II adenoid hypertrophy was 2.285 times higher than that in individuals with grade I adenoid hypertrophy, and 3.006-fold or 3.554-fold higher than for individuals with grade III and IV adenoid hypertrophy, respectively. Relative to grade I tonsillar hypertrophy, the risk of OSAS in individuals with grade II, III, and IV tonsillar hypertrophy was increased by 1.478, 1.551, and 1.631, respectively. This is consistent with other studies demonstrating that larger the adenoids and tonsils are more prone to OSAS (10, 24). While the study found that adenoid hypertrophy and tonsilar hypertrophy were not significantly associated with OSAS severity. And our study further confirmed that the tonsil size have weak relationship with OSAS severity, and adenoid sizes were no associated with pediatric OSAS. This is consistent with work published by Nolan et al. (25) which pointed out that the association between subjective pediatric tonsil size and objective OSAS severity is weak at best. Toros et al. (26) also failed to detect a link between tonsil size and objective OSAS severity. The study also revealed that the adenoid and tonsil sizes were no associated with OAHI. This is consistent with work published by Hwang et al. (27) which pointed out that Tonsil/adenoid size did not predict the severity of AHI. But Kljajic et al. (28) determined that tonsil size and adenoid size were significantly correlated with AHI. Chandra et al. (29) also found AHI to be positively associated with tonsil grade and adenoid size, and further found tonsil grade to be positively associated with AHI in toddlers, pre-schoolers, school-aged children, and adolescents, while adenoid size was positively related to AHI in toddlers, pre-schoolers, and school-aged children, but not in adolescents. As such, the use of adenoid and tonsil size is not recommended as a metric for classifying OSAS severity (1). In children that fail to exhibit significant improvements in symptoms following adenoidectomy and tonsillectomy, other causes of OSAS may also exist that require further study and elucidation. The factors described below may account for children whose symptoms do not completely improve.

Several studies have emphasized the importance of obesity as a risk factor for OSAS (8, 11, 15, 30), with some evidence suggesting that it is the primary factor related to the persistence and aggravation of childhood sleep disorders (31), consistent with an observed positive correlation between obesity and OSAS severity (32). While some reports have found obesity to be independently related to the risk of OSAS (15, 16), this finding was not replicated in the present study, and the relevance of these risk factors remains controversial. However, this study revealed a relationship between obesity and OSAS severity, suggesting that obesity may play a role in the increased risk of OSAS as reported by Bachrach et al. (15). While obese individuals may not exhibit OSAS, obesity can nonetheless contribute to pre-vertebral soft tissue hyperplasia of the upper airway, pharyngeal airway stenosis, and increased airway closure resistance, aggravating extant OSAS. Weight loss has been shown to be an effective treatment that can alleviate OSAS (33). The effective prevention of obesity in children may thus reduce the occurrence of pediatric OSAS.

Apnea-hypopnea index values have been found to be significantly correlated with environmental exposure to tobacco smoke, underscoring the potentially deleterious effects of certain environmental factors in the context of pediatric OSAS and emphasizing the importance of avoiding smoke exposure to reduce the severity of this condition (34). Similarly, tobacco smoke has been linked to increases OSAS incidence in children (19), with such exposure being independently associated with the risk of snoring among pre-school-aged children (20). It is possible that children exposed to passive smoking suffer from nicotine withdrawal during sleep, thus causing disturbances in normal sleep processes (35). In this study, we found a family history of passive smoking to be associated with pediatric OSAS risk, although it was not an independent risk factor for this condition. As such, parents should be encouraged to quit smoking and to otherwise protect their children from exposure to tobacco smoke as a means of protecting against pediatric OSAS.

A growing body of evidence supports the role of familial factors in OSAS pathogenesis. For example, tonsillar hypertrophy rates are higher in children whose parents were also affected by this condition (36). We similarly found that parental history of snoring was a risk factor associated with pediatric OSAS incidence. Some reports have suggested a higher risk of sleep apnea for children whose siblings are affected by this condition, consistent with a genetic component to sleep apnea incidence (37). As such, clinicians should ask the parents and siblings of affected patients whether they exhibit similar symptoms when taking a medical history in order to provide them with appropriate early treatment.

Here, we found allergic rhinitis to be a risk factor related to pediatric OSAS incidence, in line with prior evidence (38). Overall, allergic rhinitis prevalence rates are estimated to be 2.12-fold higher among children with sleep disorders relative to children without such disorders, with an estimated 45.2% of pediatric OSAS patients suffering from allergic rhinitis (39). While allergic rhinitis prevalence is closely related to the incidence of sleep disorders, no link between rhinitis and increased AHI severity has been reported (40). However, our study detected a correlation between allergic rhinitis and OSAS severity. Elevated levels of inflammatory mediators such as histamine, cysteine leukotriene, and interleukins in patients with allergic rhinitis can aggravate nasal obstructions leading to increased upper airway resistance, which can then result in sleep disturbances in OSAS patients (41). We did not find allergic rhinitis to be an independent risk factor associated with OSAS incidence in our study population. In previous reports, asthma has been identified as a risk factor for OSAS, with an estimated 19–60% of individuals with non-severe asthma and 95% of individuals with severe asthma exhibiting OSAS (42). In line with such reports, we found asthma to be a risk factor related to pediatric OSAS incidence, although such findings are not universal (16). Active treatment is needed to reduce the occurrence of OSAS in children with allergic rhinitis and asthma.

Pre-term birth has previously been linked to a higher risk of OSAS (43). Breastfeeding has been shown to be positively correlated with OSAS incidence, and breastfeeding rates are higher for parents with a lower education level as compared to patients with a higher education level (11). However, no correlations were detected between any of these factors and pediatric OSAS incidence in the present study.

There are certain limitations to this study. For one, this was a retrospective single-center analysis, and it is thus potentially susceptible to measurement bias, selection bias, and recall bias. In addition, the diagnostic criteria used for OSAS in the present study are specific to OSAS guidelines for Chinese children, and caution is thus warranted when extrapolating these results to other study populations.

## Conclusions

In summary, obstructive sleep apnea in children is a condition with a multi-factorial etiology. The results of this study demonstrate that obesity, family passive smoking, a family history of snoring, allergic rhinitis, asthma, tonsil hypertrophy, and adenoid hypertrophy are important risk factors associated with pediatric OSAS. Of these factors, adenoid hypertrophy and tonsil hypertrophy were found to be independently associated with the risk of pediatric OSAS, with OSAS incidence increasing with the size of the adenoid and tonsil, but the severity of OSAS is not parallel related to the adenoid or tonsil size. In clinical settings, for children exhibiting symptoms such as snoring or mouth breathing, it is important that these risk factors be taken into consideration, with a particular focus on adenoid and tonsil hypertrophy, and appropriate active clinical interventions should be administered with attention being paid to patient tonsil and adenoid size, but adenoid and tonsil size is not recommended as a measure of OSAS severity.

## Data Availability Statement

The original contributions presented in the study are included in the article/supplementary material, further inquiries can be directed to the corresponding author/s.

## Ethics Statement

Written informed consent was obtained from the minor(s)' legal guardian/next of kin for the publication of any potentially identifiable images or data included in this article.

## Author Contributions

LX collected data and wrote the article. SS and JL statistical analysis and finished statistical analysis. YJ and YS continued to check data and article. LD proposed ideas and finished project administration. All authors have read and approved the final manuscript.

## Conflict of Interest

The authors declare that the research was conducted in the absence of any commercial or financial relationships that could be construed as a potential conflict of interest.

## Publisher's Note

All claims expressed in this article are solely those of the authors and do not necessarily represent those of their affiliated organizations, or those of the publisher, the editors and the reviewers. Any product that may be evaluated in this article, or claim that may be made by its manufacturer, is not guaranteed or endorsed by the publisher.
